# Development and In Vitro Characterization of Light Responsive Zinc‐Based Nanoparticles Embedded in Collagen Sheets Intended for Wound Care Oriented Applications

**DOI:** 10.1002/mabi.70188

**Published:** 2026-04-27

**Authors:** Nunzia Gallo, Simona Bettini, Alessia Nito, Giorgia Iaconisi, Francesca Russo, Alessandra Quarta, Loredana Capobianco, Ludovico Valli, Antonio Pennetta, Giuseppe Egidio De Benedetto, Alessandro Sannino, Luca Salvatore

**Affiliations:** ^1^ Department of Engineering for Innovation University of Salento Lecce Italy; ^2^ Typeone Biomaterial Srl Lecce Italy; ^3^ Department of Biological and Environmental Sciences and Technologies University of Salento Lecce Italy; ^4^ Consiglio Nazionale delle Ricerche Institute of Nanotechnology Lecce Italy; ^5^ Institute of Atmospheric Sciences and Climate ‐ ISAC‐CNR Lecce Italy; ^6^ Department of Cultural Heritage University of Salento Lecce Italy; ^7^ Department of Experimental Medicine University of Salento Lecce Italy

**Keywords:** antibacterial scaffolds, equine collagen, nanoparticles, type I collagen, wound dressings

## Abstract

Bacterial infections are one of the most critical issues in hard‐to‐heal wounds that delay healing. The use of dressings able to both stimulate tissue regeneration and limit bacterial proliferation would reduce post‐operative therapies and hospitalization costs. To this, antibacterial wound dressings have been developed. Among antibacterial agents, Zinc and Silver‐based nanoparticles (NPs) are the most commonly used for their well‐known safety profile. Among biomaterials, collagen is recognized as the gold standard for wound dressing manufacturing because of its unique pro‐regenerative properties. With the aim of improving current wound dressings efficacy, a collagen‐based device with light‐responsive antibacterial properties was developed and in vitro characterized. A fibrillar type I collagen from horse tendon doped with patented Zinc‐based NPs was employed for the manufacturing of thin sheets. After the analysis of the chemical‐physical properties of collagen‐based sheets, the preliminary evaluation of their antibacterial efficacy confirmed their effectiveness and light‐responsiveness. The developed innovation would make it possible to in situ control the bacterial population and to reduce healing times and related costs.

## Graphical abstract

Light‐responsive Zinc‐based nanoparticles are integrated into collagen sheets to create multifunctional wound dressings. The system preserves collagen structure, transparency, and permeability while enabling on‐demand antibacterial activity against *E. coli* and *S. aureus*. This approach offers a promising strategy to locally control infections and accelerate healing in chronic wounds.

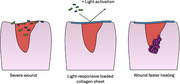

## 1 Introduction

The wound healing process occurs through a cascade of events. In normal conditions, this process happens physiologically. In some cases, natural healing processes are hindered, resulting in hard‐to‐heal, chronic wounds. In these cases, bacterial infections are one of the most critical issues that additionally delay tissue healing [1]. The risk factors associated with the development of infections at the injured site depend on the disease type and extent, adopted surgical procedure, patient's co‐morbidities, and pre‐existing infections. Currently, prevention consists of the superficial removal of microorganisms and the reduction of bacterial proliferation during the surgical procedure and in the post‐operative oral administration of antibiotics [2]. The failure of the pharmacological treatments leads to severe consequences, the most frequent outcomes of which are patient death or permanent disability. Thus, preventive strategies for infections are necessary.

The use of wound dressings able to simultaneously stimulate tissue regeneration and limit bacterial proliferation would reduce the costs for post‐operative therapies, hospitalization, morbidities, and mortality, and increase the success rates of surgical procedures [3]. To this, antibacterial biomaterial‐based formulations have been developed and extensively applied to protect the body from bacterial infections and support injured tissues regeneration [1, 3, 4]. Antibacterial wound dressings should act as physical barriers against exogenous microbial invasion, maintain a moist environment to promote wound healing, and reduce bacterial proliferation. Beyond antiseptics and antibiotics, nano‐sized technologies were used. Among them, Zinc, Silver, and Copper‐based NPs were the most commonly used [5, 6, 7].

Among biomaterials, collagen is one of the most widely used natural polymers in the healthcare‐related sector for its unique biological properties [8, 9, 10]. Collagen matrices perform a multifaceted role in the wound‐healing cascade, providing both immediate stabilization and long‐term regenerative support [11, 12, 13, 14]. Upon application, they facilitate hemostasis by promoting platelet chemotaxis and subsequent clot formation [15]. This interaction triggers the release of essential growth factors, which orchestrate the early inflammatory phase. Concurrently, the matrix's high absorptive capacity effectively manages wound exudate, maintaining the moist microenvironment necessary for repair. Beyond these initial stages, collagen serves as a bioactive scaffold that directs cellular infiltration and proliferation, ultimately guiding angiogenesis and the deposition of a functional extracellular matrix. Collagen implication in wound healing and its clinical efficacy has been detailed in an earlier study [16].

In the last decade, even more collagen‐based products enriched with antibacterial agents have been developed to enhance collagen dressing efficacy [17]. In particular, several antimicrobial compounds have been added, such as antibiotics (i. e., ciprofloxacin [18], gentamicin [19, 20, 21], metronidazole [22]) or nanoparticles (i. e., Zinc‐ [23, 24, 25], Silver‐based [26]). Indeed, because of their efficacy, several products enriched with antibacterial agents are actually commercially available for the treatment of chronic wounds, mostly with gentamicin [19, 20, 27] or silver [28].

In these circumstances, with the aim of improving the efficacy of existing wound dressings, an advanced wound dressing has been developed in this work. In particular, a collagen‐based sheet with light‐responsive antibacterial properties was proposed. The material was conceived to be used as a thin film for severe wound healing or as a surface coating on already existing devices. However, other applications, including 3D porous sponges or 2D electrospun nanofibrous matrices, are not excluded. Thus, type I collagen isolated from *Equus caballus* tendon was selected for its numerous advantages [29] and doped with three types of patented NPs at two concentrations. The developed NPs basically are ZnO‐based nanostructures decorated with a 10 nm silica shell and 20 nm Ag nanoparticles (patent number: EP3930891A1) [30], creating a composite system with enhanced, synergistic antibacterial effects. ZnO‐based nanostructures have attracted considerable interest due to their combination of optical responsiveness, chemical stability, and intrinsic antimicrobial activity. When exposed to ultraviolet or visible radiation, ZnO can generate reactive oxygen species (ROS), a process that contributes significantly to its biocidal performance [31]. The activity of ZnO can be further enhanced by functionalizing ZnO with additional functional elements. In particular, by coupling ZnO with silver (Ag) nanoparticles. Ag nanoparticles are known for their plasmonic properties, which can enhance antibacterial activity through localized surface plasmon resonance (LSPR) effects [32], as well as the release of silver ions that disrupt bacterial cell membranes [33]. Moreover, light, particularly UV or visible light, serves as an inexhaustible and fascinating trigger for these nanostructures, offering a sustainable and non‐invasive support in some therapeutic application including the management of infections and tissue repair [34]. In the present work, the focus was not on mechanistic photophysical investigation but rather on evaluating whether a detectable difference in antibacterial behavior could be observed under routine laboratory ambient‐light conditions compared with dark conditions.

Based on the current state of knowledge, this is the first investigation on the development, characterization, and in vitro functional evaluation of collagen sheets enriched with light‐responsive NPs. With the objective of taking a step toward the translational validation of biomaterials intended for wound care applications, the primary aim of the present study was the development and the preliminary in vitro validation of a light‐responsive, nanoparticle‐enriched collagen sheet prototype, with a specific focus on material engineering, physicochemical characterization, cytocompatibility, and antibacterial performance under controlled conditions. The efficacy was preliminarily assessed against a gram‐negative and a gram‐positive bacterium (*Escherichia coli* and *Staphylococcus aureus*, respectively), which are two of the most predominant organisms implicated in the initial stages of chronic wound formation [1]. This innovation might lead to the creation of fully biocompatible and biodegradable products that retain the antibacterial properties during their tunable degradation process, allowing for an in situ control of the bacterial population, the reduction of the healing times, and the increase of the recovery quality. The presence of light‐responsive NPs can therefore be very useful in thwarting bacterial infections in the long term simply by exposing the injured site to sunlight. Indirectly, as a consequence of a more effective antibacterial treatment over time, antibiotics and painkillers administration would be reduced, besides the need for resources, specialized personnel, and laboratory tests. Moreover, it would also reduce antibiotic resistance, which in turn would translate into reduced morbidity and mortality. Thus, the coupling of antibacterial NPs with a highly biocompatible, biodegradable, and bioactive biomaterial should create a favorable environment for infected wounds healing.

## 2 Materials

Horse tendon–derived type I collagen (Coll), supplied as dry flakes, was provided by Typeone Biomaterials s.r.l. (Calimera, Lecce, Italy). ZnO (Z), ZnOAg (ZA), and ZnOSiAg (ZSA) NPs were produced following a patented process of Typeone Biomaterials s.r.l. (patent number: EP3930891A1) and provided as dry powder. The Millipore Milli‐U10 water purification facility from Merck KGaA (Darmstadt, Germany) was employed for distilled water production. Bicinchoninic acid (BCA), bovine serum albumin (BSA), phosphate buffer saline (PBS), thiazolyl blue tetrazolium bromide (MTT), dimethyl sulfoxide (DMSO), and all other chemicals used for this study were purchased from Merck KGaA.

## 3 Methods

### 3.1 Coll/NPs Sheets Development

Coll/NPs sheets were manufactured through air‐drying, as detailed in a previously published work, with some modifications [29, 35]. Type I collagen in the form of dry flakes was dispersed in 0.5 m acetic acid (8 mg/mL) for 12 h under constant stirring at 10 ± 5°C. The resulting dispersion was processed at 8000 rpm for 20 min at 4°C with an IKA T25 Digital ultra Turrax homogenizer (Werke GmbH and Co. KG, Staufen im Breisgau, Germany) to achieve a uniform suspension [36]. Subsequently, the three types of ZnO‐based NPs (i. e., Z, ZA, ZSA) were added to the collagen slurry at a concentration of 0.01% and 0.001% (w/v). Each formulation was further homogenized four times at 10,000 RPM for 2 min (temperature was kept below 10°C), four times. The ZnO‐based NPs enriched collagen suspensions (Coll/Z, Coll/ZA, Coll/ZSA) were degassed under vacuum, casted in Petri dishes, and allowed to fully dry under laminar air flow for 7 days at room temperature [35]. The obtained collagen sheets were physically crosslinked by applying a dry heat treatment (DHT), performed at 121°C for 72 h under vacuum [36]. All samples underwent dry‐heat sterilization at 160°C for 2 h under vacuum [37].

### 3.2 Collagen Integrity Evaluation

Poly‐acrylamide gel electrophoresis under denaturing conditions with sodium dodecyl sulphate (SDS‐PAGE) was executed to assess collagen molecular weight variations after the sheet production process. The 8% resolving gel (with a 5% stacking gel) was hand‐cast (37.5:1 acrylamide/bisacrylamide ratio). Coll/NPs samples (10 mg) were immersed in 0.5 m acetic acid and minced. Then, a 2X standard Laemmli buffer enriched with 2 m Urea was added to each sample [38]. After 50°C for 1 h, samples were centrifuged (10000 g, 1 min) and loaded in the gel wells. Native type I collagen was provided as a control [35]. The electrophoretic run was carried out at 70 V for 20 min and then at 130 V for 2 h. After electrophoresis, the protein bands were revealed by means of a previously optimized Coomassie‐based method [36] and acquired.

### 3.3 Chemical Interactions

The chemical interactions between the fibrous protein and the ZnO‐based NPs were investigated by Fourier‐transform infrared spectroscopy (FT‐IR) by means of FTIR‐6300 from Jasco GmbH (Pfungstadt, Germany). Briefly, 1 × 1 cm Coll/NPs sheets absorption spectra were acquired from 4000 to 400 cm^−1^ at a resolution of 4 cm^−1^ (64 scans). Data were compared by plotting data with the Origin software from OriginLab Corporation (Northampton, MA, USA) [36, 39]. A total of three replicates per sample type were subjected to scanning

### 3.4 Wettability

The substrate's water‐retention capability was gravimetrically evaluated according to a previously optimized protocol [40]. Briefly, dry Coll/NPs sheets of 1 × 1 cm were weighted (*Wd*) before being incubated in 1 mL of 0.01 m PBS. At prefixed time points, wet samples weight was acquired (*Ww*). The swelling degree (*SD%*) was determined as:

(1) $$SD\%\;=\frac{W_{w}-W_{d}}{W_{d}}\;100$$

The test was performed on three independent samples for each sample type. Moreover, sheet's surface properties were evaluated by the sessile drop method (10 µl on each sample type, three times) by mean of a FTA1000 analysis system (First Ten Angstroms, Newark, NJ, USA) [36].

### 3.5 Transparency

The substrate's optical performances were spectrophotometrically evaluated. In particular, round‐like Coll/NPs samples (radius = 4 mm) were casted on the bottom of a 96‐well multiwell plate and hydrated with 0.1 mL of 0.01 m PBS. After 2 h, the excess of PBS was removed, and the transmittance of swelled samples was acquired by means of an Envision Multimode Plate Reader (PerkinElmer Inc., Waltham, Massachusetts, USA). Each sample was tested using three independent replicates.

### 3.6 Mechanical Properties

The stress–strain properties of hydrated Coll/NPs sheets were evaluated in terms of tensile test, suture retention, and skin adhesion test. A ZwickiLine universal testing machine (Zwick/Roell, Ulm, Germany)—loading cell: 1 kN—was used.

As regards tensile tests, Coll and Coll/NPs samples of 5 × 20 mm (previously fully swollen in 0.01 m PBS), were tested under displacement‐control mode, up to rupture. For constitutive bond determination, loading proceeded at a displacement rate of 0.1 mm/s (0.1 N preload) [36]. The Young modulus (E), the stress at break (𝜎max) and the strain at break (𝜀r) were registered [41]. In particular, E was extracted by fitting the linear portion of the stress–strain curve observed at low deformations (1%–5%) [35, 36]. Triplicate measurements were performed for each sample.

For the suture anchorage tests, a 5–0 suture thread (VICRYL Polyglactin 910, Ethicon, Johnson & Johnson International, New Brunswick, NJ, USA) was placed 2 mm from the shorter border of Coll/NPs samples. The load was applied with a speed of 150 mm/min (0.1 N preload), according to ISO 7198. Suture retention strength was quantified as the peak stress value obtained from the stress–strain curve. The experiments were executed six times for each sample.

### 3.7 Permeability to Proteins

Mass transport is fundamental for cell processes and thus for tissue regeneration. The permeability to protein was evaluated by protein diffusion studies across the membranes. Since diffusion depends on various factors like molecules' molecular weight, test temperature, pressure, diffusion medium, and membrane characteristics [42, 43, 44], two model molecules were chosen and tested for diffusion studies across several membranes in a physiological‐like environment, at constant temperature (25°C) and pressure (1 atm). In particular, lysozyme (14 kDa) and BSA (66 kDa) were chosen.

Each collagen‐based substrate was fixed between the two compartments of the diffusion chambers. The acceptor chamber was charged only with PBS 1X (pH 7.4), and the receiver chamber was loaded with 1 mg/ml of protein in PBS 1X (pH 7.4). The proteins' diffusion across the collagen sheets was monitored by quantifying their content in the receiver chamber at prefixed time points (2, 4, 8, 15, and 30 days) by means of the BCA kit [45]. Permeation coefficients were derived from the equation [42, 43, 44]:

(2) $$ln\;\left(1-\frac{2C_{t}}{C_{0}}\right)=-\frac{2AU}{V_{c}L}t$$

where *C_t_* denotes the protein content in the receiver chamber at the prefixed time point *t*, *C_0_* is the protein concentration at *t* = 0 in the receiver chamber, *A* is the diffusion area, *U* describes the permeability coefficient, *V* corresponds to the chamber volume, and finally *L* represents the sheet thickness. Based on this equation, plotting:

(3) $$-\frac{V_{c}L}{2A}ln\left(1-\frac{2C_{t}}{C_{0}}\right)\;versus\;t$$

allowed to determine U as the slope of the interpolated linear fitting. The diffusion coefficient (D) was determined as U/K (Equation 3). Here, K corresponds to the partition coefficient, defined as the ratio of the solute content in the Coll/NPs sheet to that present in the chamber.

(4) $$D=\frac{U}{K}\;$$

Thus, K was determined by means of the depletion method that consisted of equilibrating a Coll sheet in 1 mL of a 1 mg/ml BSA or lysozyme solution sheet. K was defined as follows:

(5) $$K=\frac{V_{s}\left(C_{0}-C_{s}\right)}{V_{m}C_{s}}$$

where V_s_ corresponds to the volume of the solution, V_m_ represents the volume of the Coll/NPs sheet, C_0_ is the initial solute content, and C_s_ is solute concentration once equilibrium is reached.

In order to correlate the protein dimensions with diffusion coefficients, protein molecular radii and self‐diffusion coefficients were also calculated. The radius of each solute (r) was approximated under the assumption that the molecules behave as spherical particles, as:

(6) $$r^{2}={\left(\frac{3V_{mol}}{4\pi N_{0}}\right)}^{2/3}$$

where V_mol_ is the molecular volume of solute, and N_0_ is Avogadro's number. The experiment was run three times for each sample type.

### 3.8 Degradation Resistance

The degradation resistance of Coll/NPs sheets was in vitro estimated in physiological‐like conditions. Three samples for each category of approximately 10 × 10 mm were weighted (*W_0_*) and incubated at 37°C in 2 mL of 0.01 m PBS, pH 7.4. At fixed time points, the degraded collagen amount (*W_t_*) was estimated by means of the colorimetric BCA assay. The degradation resistance (R%) was evaluated as:

(8) $$R\%=\left(1-\frac{W_{0}-W_{t}}{W_{0}}\right)\;\;100$$

Along with the Coll/NPs sheets degradation resistance evaluation, NPs release over time was assessed by inductively coupled plasma mass spectrometry (ICP‐MS). A 500 µL aliquot of each sample was diluted 1:5 with 2% HNO_3_. Subsequently, 100 µL of a 5 mg/L yttrium solution, used as an internal standard, was added to the samples before analysis. The monitored isotopes were 64 Zn and 89Y in KED mode, and an external calibration curve was used for zinc quantification. The NPs release (NPrel%) was calculated as the NPs recovered in the testing medium % using the following equation:

(9) $$NPrel\%\;=\left(1-\frac{NP_{0}-NP_{t}}{NP_{0}}\right)\;\;100$$

where *NP_t_* is the mass of NPs released from the Coll/NPs samples during time (*t*), and *NP_0_* is the total quantity of NPs loaded in the Coll/NPs sheets. Triplicate assessments were performed for each sample type and time point.

### 3.9 Antibacterial Efficacy

The antibacterial ability of Coll/NPs sheets was checked against the Gram–negative bacterium *E. coli* (ATCC 25922) and *S. aureus* (ATCC 29213) according to the AATCC Standard Test Method 100 (1993) from the AATCC Technical Manual, with some modifications [46]. The bacterial strains were propagated at 37 ± 1°C in Luria–Bertani (LB) broth (1% tryptone, 0.5% yeast extract, 1% NaCl) overnight. After being adjusted to 4 × 10^6^ colony forming units (CFU)/mL with 10 mL saline containing 0.1% Tween 80 and 0.01% Triton X–100, 10 µl of the microbial test inoculum was added to sterilized Coll/NPs sheets, samples of 1 × 1 cm.

Antibacterial experiments under illuminated conditions were performed under ordinary laboratory ambient light generated by standard neon lamps. Samples were exposed for the same duration as the corresponding dark‐condition experiments (1 h), in order to allow a direct comparative assessment. The illuminated condition was intended to reproduce routine indoor visible‐light exposure in a laboratory setting and was used as a preliminary proof‐of‐concept comparison rather than as a quantitative photophysical investigation. No dedicated monochromatic light source or irradiance calibration was employed. Dark controls were conducted in parallel under otherwise identical experimental conditions. After 1 h of incubation (humidity of >90%) in hermetically sealable vials, microorganisms were recovered by adding 10 mL of saline containing 0.1% of Tween 80 and vortexing for 1 min. The incubation time was consistent with standardized surface antibacterial testing procedures (in line with the AATCC Standard Test Method 100, 1993). Then, 10 µl of suspension was recovered and plated on LB–agar previously casted in Petri dishes. After 24 h of incubation at 37 ± 1°C (relative humidity of > 90%) the number of CFU/ml was determined. According to the AATCC100, the microbial titer reduction (MTR%) was calculated as:

(10) $$MTR\%\;=\frac{C_{i}-C_{f}}{C_{i}}\;100,$$

where *C_i_* is the bacterial concentration (CFU/ml) recovered from the inoculated untreated fabric, and *C_f_* is the bacterial concentration (CFU/mlL recovered from the inoculated antibacterial fabric after 1 h of incubation. Coll sheets without NPs were used as a control. The test was performed in quadruplicate for each sample type.

### 3.10 Proliferation and Compatibility Assay

The MTT assay was performed to assay the proliferation of the embryonic mouse fibroblast cell line, NIH/3T3, grown over the Coll/NPs sheets. In detail, Coll/NPs sheets were laid in each well of a 48‐well plate, and cells were seeded on them at a density of 5 × 10^3^. The samples were incubated at 37°C in a humidified atmosphere for 24 and 96 h. After incubation, the culture medium was replaced with serum‐free medium supplemented with 2 mg/mL MTT, before being incubated for 2 h at 37°C. Then, the reactive solution was removed, and the resulting formazan crystals were solubilized in DMSO. Cell proliferation was quantified by calculating the absorbance ratio at 570 nm between the measurements of each sample and compared to control samples (cells cultured directly on the plastic surface of the well plates). All tests were conducted in triplicate for each sample type.

### 3.11 Cell Imaging

To assess cellular adhesion on collagen sheets, NIH/3T3 cells were seeded on Coll/NPs sheets as described above. After 24 and 96 h, cells were fixed in paraformaldehyde (4% in 0.01 m PBS) and stained with DAPI (1 µg/mL) and Phalloidin‐TRITC (50 µg/mL) to label cell nuclei and cytoskeleton actin filaments, respectively. Afterwards, cells were visualized through a fluorescence microscope (EVOS M7000, Imaging System). To correlate the proliferation data to imaging, cell nuclei in each sample were quantified by ImageJ (National Institute of Health, Bethesda, MD, USA). Each sample type was quantified in three. Optical imaging of the cells grown over the collagen sheets was also carried out using an Axio Imager A2m equipped with an Axiocam MRc 5 (Carl Zeiss, Oberkochen, Germany).

### 3.12 Statistical Analysis

The Student's *t*‐test was employed to assess statistical significance. All measurements were reported as mean ± standard deviation, and a *p*‐value < 0.05 was considered to indicate statistical significance.

## 4 Results

### 4.1 Collagen Integrity Evaluation

**FIGURE 1:**
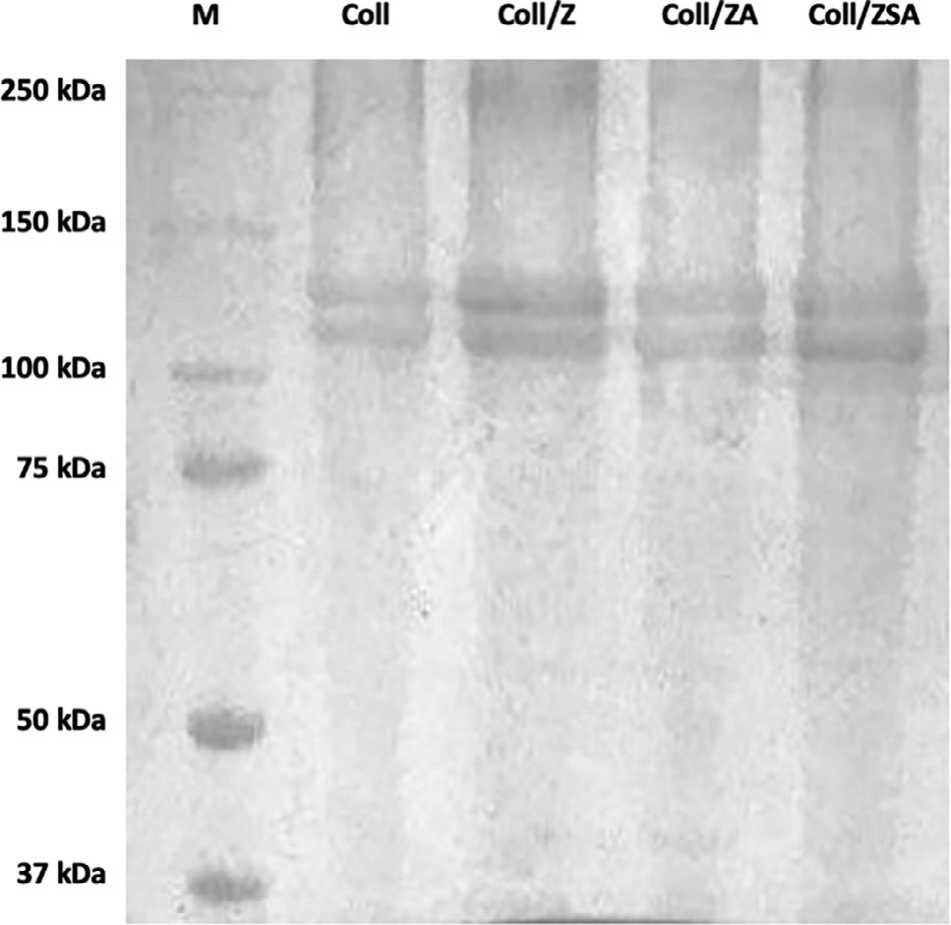
Electrophoretic pattern of Coll/NPs sheets in comparison with standard proteins (M), and control (type I collagen; Coll).

To evaluate both the protein composition and the potential impact of the sheet‐processing steps on collagen molecular integrity, SDS‐PAGE analysis was performed. The band patterns obtained for the Coll/NPs sheets, compared with standard markers, are presented in Figure 1. The protein pattern of type I collagen from equine tendon, which is the raw starting material used for the sheets' development, was provided as a reference of the degree of integrity and purity of the material [35]. Consistent with type I collagen behavior, the gel revealed two distinct bands associated with the α1(I) and α2(I) chains, appearing near 140 and 120 kDa, respectively. All analyzed samples were found to be composed of the same bands, suggesting that the employed production process did not alter the collagen molecular weight. Moreover, the absence of band shift supported the hypothesis of non‐covalent interaction between collagen and ZnO‐based NPs.

### 4.2 Chemical Interactions

FTIR spectroscopy was performed to investigate protein secondary structure and its chemical groups. The FTIR spectra of Coll/NPs were compared to those of Coll sheets without NPs (Figure S1). The characteristic amide bands (I, II, and III) of collagen were present. The amide I band (1630–1660 cm^−1^) corresponds to C═O stretching vibrations, primarily associated with the protein's secondary structure, including β‐sheets and α‐helices. The amide II band (1530–1560 cm^−1^) is attributed to the stretching of the C─N and to the in‐plane bending of the N─H group of the amide linkage. The amide III band (1200–1220 cm^−1^) corresponds to N─H bending vibrations. The FTIR spectra revealed that the addition of ZnO‐based NPs did not induce notable alteration in the position of the amide bands, suggesting that the collagen secondary structure remains largely intact [47, 48]. However, slight variations in peak intensity, particularly in the amide I and amide II regions, may indicate weak interactions between ZnO‐based NPs and collagen fibers [24, 49]. These interactions could be due to hydrogen bonding or electrostatic forces between the nanoparticle surface and collagen functional groups [24, 47, 48].

### 4.3 Water Retention Ability

**FIGURE 2:**
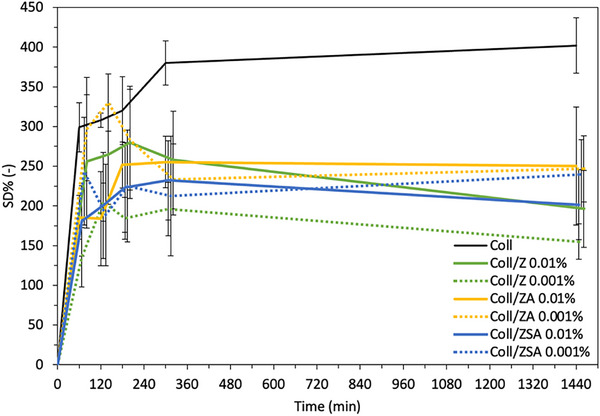
Swelling degree profiles of Coll/NPs sheets evaluated alongside that of Coll sheets. Results are represented as mean ± SD (*n* = 3).

Coll/NPs sheets' ability to absorb and retain water is of fundamental importance for wound dressing applications. The SD% of Coll/NPs samples was reported in Figure 2. Compared to the Coll sheet, Coll/NPs sheets revealed to have a SD% value of about 200%–250%. The reduction of the SD% value of about 1.5‐fold could be clearly attributed to the presence of NPs and to the interactions between them and collagen fibers. However, no statistically significant differences were revealed among samples (*p* > 0.05), suggesting that NPs type and concentration did not influence collagen sheets' water retention ability.

The evaluation of Coll/NPs sheets surface properties gave back contact angles values of about 80–90°, with no statistically significant differences among samples (*p* > 0.05), suggesting that the slight hydrophobic character of sheets is due to the collagenous component.

### 4.4 Transparency

**FIGURE 3:**
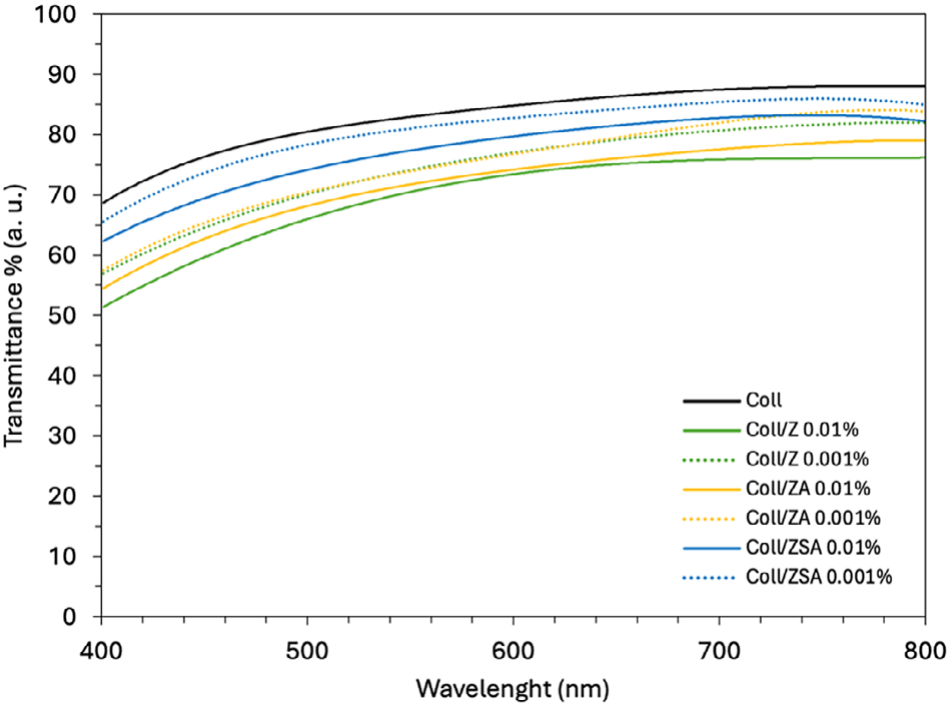
Representative transmittance curves of Coll/NPs sheets in comparison with Coll sheets in the UV–vis range.

Optical properties of Coll/NPs sheets are of fundamental importance when prototypal devices application area and potential should be defined. Transparency of Coll/NPs sheets in comparison with NPs free Coll sheets was reported in Figure 3. Coll sheet light transmittance was found to be of about 70%–80%, according to literature about collagen‐based thin substrates [50, 51]. Generally, the light transmittance of all sheets was found to increase with the wavelength. A plateau was reached from about 600 nm, suggesting a constant transparency degree in the range of visible light. Transparency values higher than 70% indicated optimal optical properties [51, 52]. Coll sheets were found to have transparency values ranging from 70% to 90%, suggesting the potential application of them for a wide range of applications, including corneal repair, dura mater repair, or for wound healing. This occurs thanks to the ability to immediately and partially recover the sight and to directly observe post‐operatively what happens in the injured site over time. The addition of NPs was found to decrease the sheet's light transmission power, in a directly proportional manner with NPs concentration. A transparency reduction of approximately 5% was observed in each Coll/NPs sheet, regardless of the NPs type. Moreover, the NPs type influenced the light properties of the sheets. Indeed, Z NPs were found to mostly reduce the sheet's light transmittance, followed by ZA and lastly by ZSA. Thus, the developed technology allowed to reduce the influence of the presence of NPs in the Coll sheets, with a reduction of the light transmittance values of about 5% in the case of 0.001% ZSA/Coll and of about 10% in the case of 0.01% ZSA/Coll. However, although reduced, the light transmittance of ZSA/Coll sheets in the range 600–800 nm was found to be higher than 80%, suggesting their acceptable level of transparency [52, 53].

The transparency value recorded for Coll sheets was found to be similar to that of bovine tendon‐derived collagen films. Indeed, uncrosslinked films manufactured from a 2.5 mg/mL slurry [53], carbodiimide crosslinked films obtained from slurries of unknown collagen concentration [52], and glutaraldehyde crosslinked film from a 10 mg/ml collagen slurry [54] were all found to have a light transparency degree in the range 70%–90% in the visible light range. However, besides the material properties, other parameters can affect the substrate's transparency, such as the thickness, the polymer concentration, the solvent used, and the crosslinking treatments applied. Indeed, our Coll sheets properties were found to be higher than those recorded on other collagen films obtained from bovine tendon too [50].

### 4.5 Mechanical Properties

Coll/NPs sheets' mechanical properties compared to NPs‐free Coll sheet are reported in Table 1. Coll sheet properties were found to be coherent with the ones reported in our works [36, 55]. Coll/NPs sheets behavior was found to be similar to Coll sheets; however, the presence of the NPs and their amount influenced their properties. Generally, the addition of NPs to Coll sheets led to matrices stiffening, visible from the increase of E and of 𝛔max and the decrease of 𝛆r%. As regards the elastic modulus, while the lowest concentration of NPs did not significantly influence it, the highest was found to be able to increase its value by about 1–2 mPa. Among NPs type, ZSA were found to induce the highest matrix stiffening, followed by ZA and Z. Instead, in the case of 𝛆r%, while the presence of NPs was responsible of the sharp decrease of its value that quite halved in all Coll/NPs samples, a not statistically significant difference was revealed among samples, suggesting that NPs type and concentration did not influence it. Conversely, the 𝛔max value was found to slightly increase in all Coll/NPs samples type compared to Coll (*p* > 0.05). Similarly, the suture retention ability of Coll/NPs sheets was found to be decreased by about 10 g, with no statistically significant differences among almost all samples (*p* > 0.05).

**TABLE 1:** Mechanical properties of Coll/NPs sheets in comparison with Coll sheets in terms of E (a), 𝛔max (b) 𝛆r (c), and suture retention strength (SR). Values reported represented mean ± SD, where *n* = 6.

Samples	E (MPa)	𝛔 max (MPa)	𝛆r %	SR (g)
Coll	2.5 ± 0.3	2.3 ± 0.4	53 ± 8	47 ± 7
Coll/Z 0.001%	2.1 ± 0.5	2.5 ± 0.3	19 ± 8	38 ± 5
Coll/Z 0.01%	2.9 ± 0.8	3.0 ± 0.4	22 ± 7	30 ± 6
Coll/ZA 0.001%	2.6 ± 0.4	2.4 ± 0.6	25 ± 7	37 ± 3
Coll/ZA 0.01%	4.4 ± 0.6	3.2 ± 0.6	18 ± 3	33 ± 5
Coll/ZSA 0.001%	3.0 ± 0.5	2.7 ± 0.2	10 ± 4	34 ± 7
Coll/ZSA 0.01%	4.6 ± 0.9	3.3 ± 0.7	9 ± 5	27 ± 4

Changes in Coll sheets mechanical properties could be ascribed not only to the presence and the type of enclosed NPs but also to the sheets production process. The performed cycles of homogenization could have impacted the sheets performances. In particular, homogenization cycles could have shortened collagen fiber length, leading to an increase of E and a decrease of 𝛆r [35]. The homogenization step could have better disassembled collagen fiber and allowed for a higher exposure of functional groups available for hydrogen interactions that could have led to the formation of stronger networks compared to NPs‐free Coll sheets. Although homogenization could have shortened fiber length and decreased the resulting sheets' properties, the homogenization step allowed for a more uniform fiber and NPs dispersion and thus to more reproducible material properties.

### 4.6 Permeability

**FIGURE 4:**
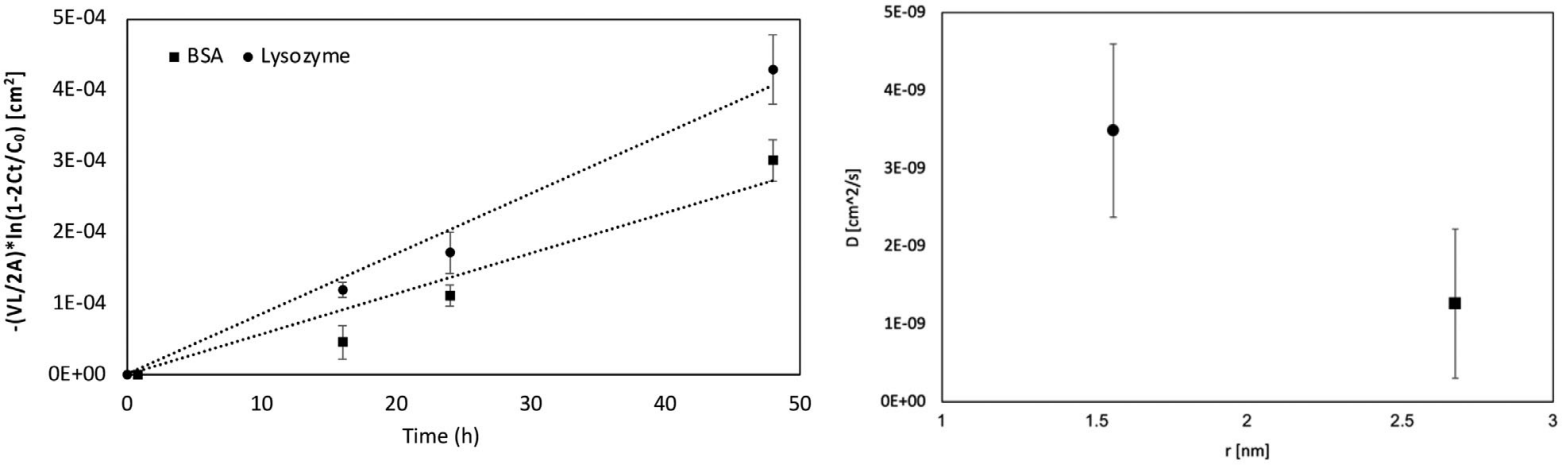
Variability of permeability coefficients of lysozyme (circle) and BSA (square) with collagen substrates on the left. The slopes represent the permeability coefficients (U) through the sheets. Relationship between diffusion coefficient (D) and protein radius on the right. Values indicated represent mean ± SD, where *n* = 3.

The permeability to protein was assessed by diffusion studies across Coll and Coll/NPs sheets. The U values of the two chosen globular proteins, lysozyme and BSA, were determined as the slope of the linear relationship between time and a value proportional to the concentration of the protein in the receiver chamber, as described in Equation (2). As shown in Figure 4, the permeability to proteins of Coll sheets was confirmed by the increasing U values over time. A similar behavior was observed for both lysozyme and BSA, with a significant influence of the protein hydrodynamic radius (lysozyme: r = 1.56 nm; BSA: r = 2.68 nm), which was found to be inversely proportional to D (*p*<0.001) (Figure 6) [44]. Thus, lysozyme was found to diffuse more rapidly across the Coll membrane than BSA. As regards Coll/NPs sheets, the presence of NPs was found not to affect Coll sheets diffusion properties (data not shown). This result could be ascribed to NPs nano size, which did not influence the pore type permeation mechanism of proteins across the Coll sheets.

### 4.7 Degradation Resistance

**FIGURE 5:**
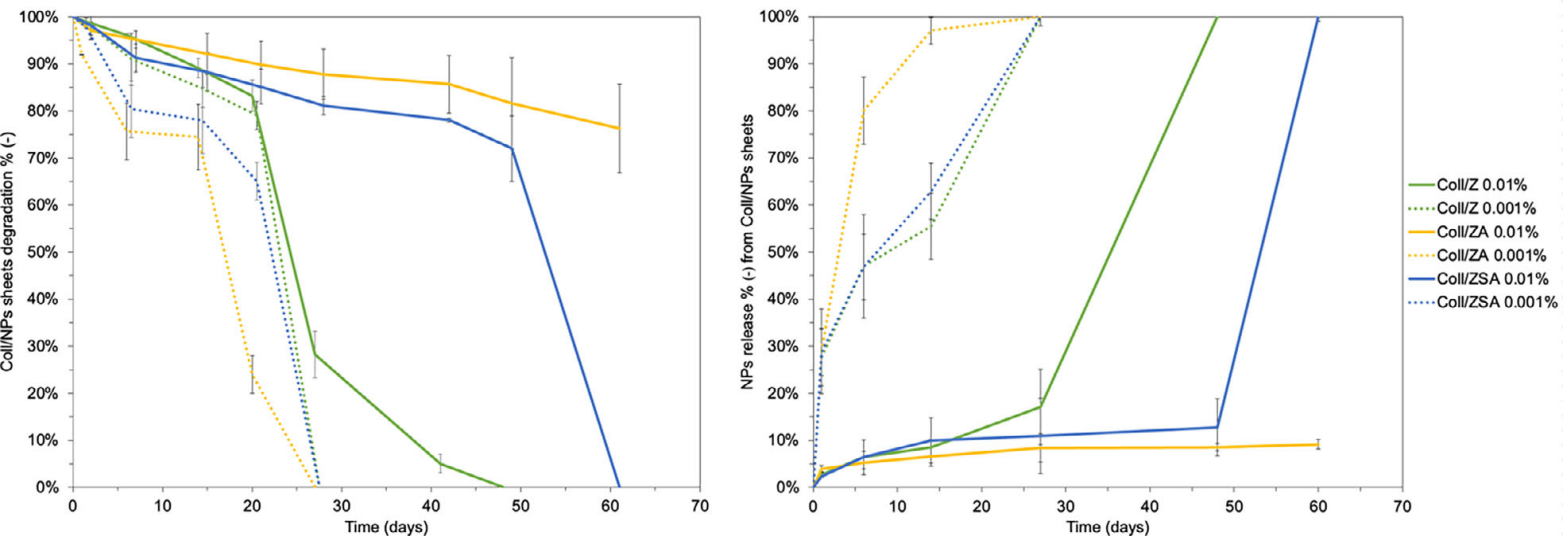
Degradation curves of Coll/NPs sheets (left) and their relative NPs release (right) up to 60 days of incubation in physiological‐like conditions. Values reported represented mean ± SD (*n* = 5).

The resistance of Coll/NPs sheets to degradation in physiological‐like conditions is one of the most important and impactful properties of medical devices, as it strongly influences their performances and applications. Degradation curves of Coll/NPs sheets were reported in Figure 5, as well as the NPrel% up to two months. The presence and the quantity of NPs significantly increased Coll sheets half‐life. Indeed, Coll/NPs sheets with a higher concentration on NPs were found to be more resistant to degradation compared to Coll/NPs sheets with the lowest concentration of NPs. Indeed, Coll/NPs 0.01% completely degraded after 50–80 days, while Coll/NPs 0.001% only after 25–30 days. Moreover, the NPs type had a strong impact. Despite data were not significative at a concentration of NPs of 0.001% (*p* > 0.05), clear differences were observed for 0.01% NPs. In particular, ZA were found to be able to increase Coll sheets stability more than the other NPs type, followed by ZSA and Z. During the degradation resistance test, also NPs release during time was also evaluated and reported in Figure 7. A burst release of NPs was registered in Coll/NPs sheets with the lowest concentration of NPs after 1 day, with a complete release after 15–20 days, in accordance with the respective Coll/NPs sheets degradation resistance. Coll/NPs sheets with 0.01% NPs were found to be able to hold NPs for a longer time. Indeed, the burst release observed for Coll/NPs 0.001% was not observed for this experimental group, beyond the NPs type. Indeed, less than 5% of NPs were released after 1 day and about 10% after 15 days. Also, in the case of Coll/NPs 0.01%, the release of NPs became higher when degradation of the Coll matrix occurred. Indeed, the complete degradation of Coll/Z and Coll/ZSA was accompanied by Z and ZSA 100% release after 40 and 55 days, respectively. Coll/ZA 0.01% were found to be the most resistant to degradation, with only a 20% of weight loss and a 10% of ZA released after 60 days of incubation in physiological‐like conditions.

### 4.8 Antibacterial Efficacy

**FIGURE 6:**
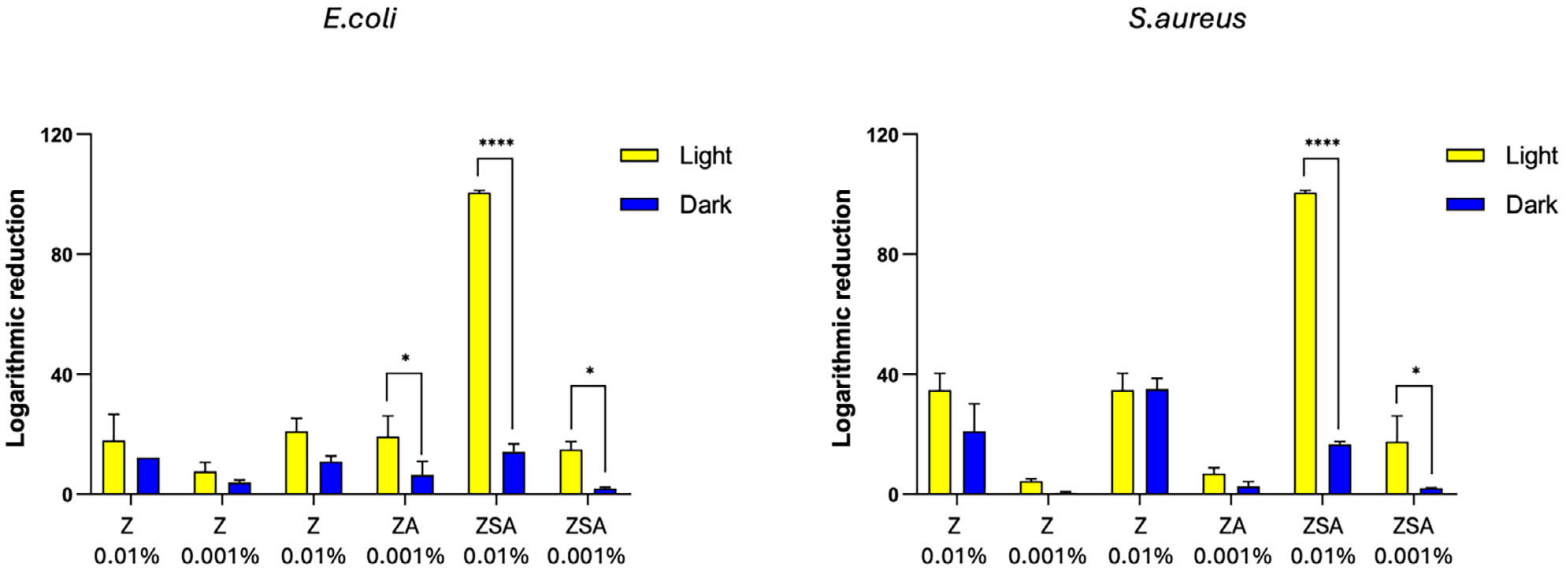
Percentage reduction of the antibacterial activity of Coll/NPs sheets in the presence of light or in the darkness, after 1 h of incubation with 4 ( 10^6^ CFU/ml *E. coli* (left) and *S. aureus* (right) suspension. Values reported represented mean ± SD, where *n* = 4.

The antibacterial activity of the Coll/NPs sheets was preliminarily assessed against E. coli and S. aureus. Coll sheets without NPs were used as control samples. As expected, no reduction of the antibacterial activity was observed. As reported in Figure 6, all substrates were able to reduce both bacterial titers. Generally, Coll/NPs seemed to be more effective against E. coli compared to S. aureus since a slightly higher bacterial log reduction was registered. In the presence of E. coli, samples incubated in the dark were found to be able to reduce the bacterial titer of 10%–30%, according to the NPs concentration. Negligible differences were observed among NPs type. Conversely, ambient light exposure of samples during the test showed the potential of patented ZSA NPs. In particular, a significant reduction of the MTR% was registered, with a complete inhibition of bacterial growth with 0.01% of ZSA. The behavior of Coll/NPs sheets in the presence of S. aureus was found to be similar to that in the presence of E. coli, since a reduction of the bacterial titer of about 10%–40% was observed, according to the NPs concentration. Also in this case, the exposure to light allowed for a complete bacterial growth inhibition with 0.01% of ZSA. These results suggested how the use of these advanced nano‐systems could limit bacterial growth by employing a lower NPs concentration and how the antibacterial power could be enhanced on demand. Thus, results represent preliminary evidence supporting the feasibility of the proposed light‐responsive system as an antimicrobial.

### 4.9 Biocompatibility

The colorimetric MTT assay was used to evaluate and compare the cytocompatibility of the Coll/NPs sheets with that of the bare NPs, and to estimate the proliferation of fibroblasts grown over the Coll sheets after 24 and 96 h incubation. The viability assay, whose results are reported in the Supplementary Information (Figure S2), shows that the NPs have an optimal biocompatibility with no effects on cell proliferation degree. Coll/NPs sheets were found to have a good biocompatibility, with a cell viability higher than 75%. The slightly lower proliferation degree should be interpreted not as cytotoxicity but as temporary slowing down of cell metabolic activity due to the presence of a biomaterial with a surface stiffness that is significantly different from that of the wells of the multiwell plate [56, 57, 58].

**FIGURE 7:**
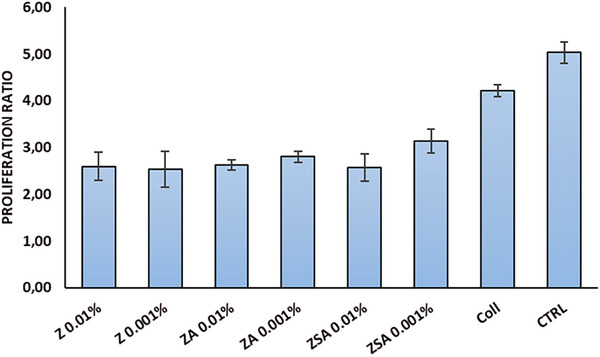
Proliferation ratio of NIH/3T3 cells seeded over Coll and Coll/NPs sheets after 96 h, estimated through MTT assay.

When the cells were grown over the Coll/NPs sheets the proliferation rate was estimated at 24 and 96 h incubation times (Figure 7). In the case of the Coll sheets, the ratio (around 4) is slightly lower than that of control cells (around 5), while in the case of Coll/NPs sheets (around 3), the values are almost all similar and lower than that of the Coll sheet. These results show the ability of the Coll sheets to support cell adhesion and proliferation on one hand, and that the presence of the NPs is likely to modify the surface topography of the sheets, thus affecting cell adhesion. Notably, the cells adhered to the Coll/NPs sheets are viable as demonstrated by the viability assay and the cell counting reported in the following section.

### 4.10 Cell Imaging

**FIGURE 8:**
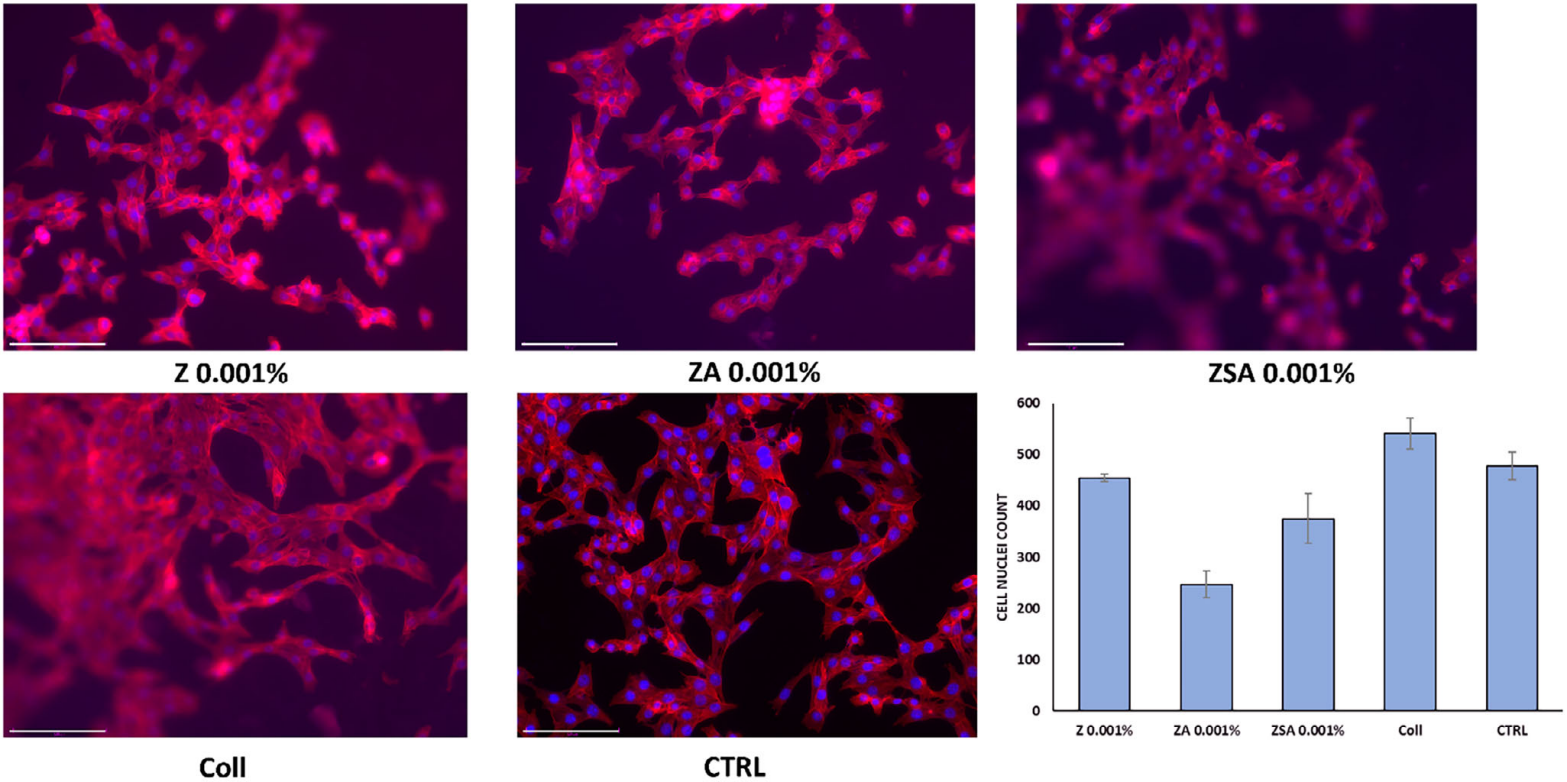
Fluorescent Images of NIH/3T3 cells seeded over Coll/NPs 0.001% and Coll sheets stained with DAPI (in blue) and Phalloidin‐TRITC (in red) after 96 h. Scale bar is 125 µm. The graph reports the cell nuclei count of Coll/NPs 0.001%, and Coll sheets compared to control samples.

To visualize cell adhesion and morphology over Coll sheets through fluorescence microscopy after 96 h incubation, NIH/3T3 cells were stained with DAPI (for nuclei staining) and Phalloidin‐TRITC (for tubulin staining). As reported in Figure 8, the cells grown on Coll sheets were abundant, indicating that the material is biocompatible and able to support cell adhesion and proliferation [36, 59]. Furthermore, the fibroblasts retained their typical elongated shape, suggesting that the presence of the sheets did not cause any changes in their morphology (Figure 8). In addition, cell nuclei counting was comparable to that of the control sample. In the case of Coll/NPs sheets the morphology of the cells was also comparable to the control sample, but the overall number of cells was lower. These data were in agreement with those of the proliferation assay.

The same experiment was performed in the case of the cells seeded and grown over Coll/NPs 0.01%, but, likely due to the higher concentration of nanoparticles, the reduced transparency of the films did not allow optimal imaging. Indeed, Figure S3 shows some images of NIH/3T3 cells incubated over Coll/NPs 0.01% for 96 h: quality and focus are poor, and the cells look blurry. Compared to the images of the cells over Coll/NPs 0.001% here the cellular shape looks more round‐like, witnessing a lower capacity to spread out on the film. Optical imaging of the cells adhered to the collagen films is also reported in Figure S4, which refers to the fibroblasts after 4 days of growth on the different types of samples. The bright field images witness the presence of a rough substrate under the cells that display the typical elongated morphology.

## 5 Discussion

The role of collagen in regenerative medicine is well‐established, owing to its ability to serve as a bioactive scaffold that closely mimics the natural extracellular matrix. As we have detailed in our previous work [16], the efficacy of collagen in promoting rapid re‐epithelialization and granulation tissue formation is fundamental to reducing healing times across various wound aetiologies. However, in complex clinical environments, the structural benefits of collagen alone are often insufficient, as bacterial contamination remains the primary barrier to successful tissue repair. Thus, there is currently a high clinical demand for intelligent dressings. Collagen‐based dressings functionalized with antimicrobial components directly address this need. By integrating antibacterial agents into the collagenous matrix, it is possible to inhibit biofilm formation while preserving the material's intrinsic properties.

In this frame, the present work demonstrates the development and preliminary validation of Coll‐based wound dressings enriched with innovative light‐responsive Z‐based NPs. The engineering of these nanostructures into Coll matrices allows to combine the regenerative potential of Coll with the antimicrobial efficacy of NPs, offering a dual‐function platform ideal for hard‐to‐heal wounds management. In particular, Coll/NPs sheets would temporally support tissue regeneration by mean of the well‐known Coll bioactive properties and would control antibacterial activity by mean of NPs, with antibacterial performance that can be modulated under light exposure. Such features can address key clinical needs in the management of chronic and hard‐to‐heal wounds, where microbial colonization often hinders physiological healing and increases the risk of complications. Specifically, in this work, light‐responsive, nanoparticle‐enriched collagen sheet prototypes were developed and systematically characterized from their physicochemical properties to cytocompatibility and antibacterial performance under controlled conditions. Thus, this study represents a critical early step toward the translational validation of biomaterials for wound care applications and the in vivo investigation.

The adopted manufacturing approach allowed to preserve the structural integrity of collagen, as demonstrated by SDS‐PAGE and FTIR analysis, and ensured favourable physical and chemical characteristics, including optical transparency, mechanical resistance, and protein permeability. The incorporation of NPs did not compromise the matrices' performance as wound dressing substrates. Although water uptake capacity and surface hydrophilicity were slightly reduced compared to Coll sheets, all formulations maintained an adequate moisture balance necessary for optimal wound healing. This suggests that the hydrophilic nature of collagen remains dominant, even after doping, and maintains the essential characteristics required for wound exudate management. Similarly, light transmittance remained above 70%, supporting potential applications in wound monitoring, ocular repair, or transparent medical barriers. These findings are crucial for applications requiring direct visual monitoring of the wound site or compatibility with other diagnostic tools. Notably, the mechanical data revealed that NPs incorporation induced a matrix stiffening effect, pronounced at higher concentrations. However, these changes remained within acceptable ranges for soft tissue applications and did not compromise the materials' handling or suture retention capabilities. Importantly, permeability to model proteins was retained in all doped scaffolds. This feature is crucial for the diffusion of nutrients, growth factors, and waste products across the dressing during tissue repair. NPs loading did not obstruct the collagen microstructure to an extent that would hinder mass transport. From a degradation standpoint, degradation kinetics of Coll/NPs sheets were significantly modulated by both NPs type and concentration. Coll/NPs sheets enriched with higher concentrations of NPs displayed extended resistance to enzymatic breakdown and a higher controlled release of NPs. The tunability of degradation resistance allows for design flexibility according to the wound severity or anatomical location. Moreover, the release kinetics of the NPs fitted well with the degradation rate of the Coll matrix. The 0.01% ZSA‐based sheets exhibited a slow and sustained degradation pattern, maintaining their structural and functional properties over extended periods. This is particularly advantageous for long‐term wound care, especially in chronic cases, where extended dressing lifespan and infection control are critical.

The most compelling feature of the developed dressings lies in their enhanced antibacterial activity. In vitro assays revealed that the Coll/NPs sheets were effective against both *E. coli* and *S. aureus*, the two most clinically relevant pathogens. Notably, Coll/ZSA sheets exhibited enhanced antibacterial activity under laboratory ambient‐light conditions compared with dark conditions, leading to complete bacterial growth inhibition under ambient light at the highest NPs concentration. It is important to clarify that the illumination used in this study consisted of ordinary laboratory ambient light generated by standard neon lamps and was intended as a preliminary proof‐of‐concept comparison rather than as a quantitative photophysical investigation. Accordingly, the present results should be interpreted as evidence of a detectable light‐associated enhancement of antibacterial behavior under routine indoor visible‐light conditions. The antibacterial activity observed under illumination is consistent with the known photoactive properties of related ZnO‐based nanostructures previously reported by our group [30]. Nevertheless, the specific mechanism underlying the light‐enhanced antibacterial effect within the collagen matrix was not directly investigated, as the study was deliberately designed to evaluate antibacterial performance under routine laboratory ambient‐light conditions. In this perspective, the use of standard indoor illumination reflects practical and easily reproducible exposure settings rather than controlled wavelength‐specific irradiation systems.

The study of films cytocompatibility evidence that both starting film and Coll/NPs display good biocompatibility. Indeed, fibroblasts adhere, grow, and spread over the films. The morphology of the cells is comparable to that of the control (fibroblasts seeded on the plastic well), and the proliferation rate up to 4 days is slightly slower in the case of Coll/NPs, likely due to the presence of the nanoparticles. The experimental data revealed a distinct dose‐dependent trade‐off between the antimicrobial and regenerative properties of Coll/ZSA. While the 0.01% ZSA concentration exhibited the most robust antibacterial efficacy, the 0.001% dosage proved superior in promoting cellular adhesion and proliferation. This divergence highlighted a critical balance: higher concentrations maximize pathogen clearance, whereas lower concentrations preserve the microenvironment necessary for tissue integration. Future investigations will focus on expanding the concentration range and performing detailed dose–response analyses to identify the optimal window for synergistic antimicrobial and regenerative performance.

Overall, this study presents an original and promising platform that integrates bioactivity, biodegradability, physico‐chemical‐mechanical compliance, and tunable antimicrobial function in a single scaffold system, potentially paving the way for next‐generation wound dressings that align with personalized and minimally invasive therapeutic approaches.

## 6 Conclusion

Collagen‐based dressings have emerged as a cornerstone in regenerative medicine due to their unique ability to actively intervene in the wound‐healing cascade. Despite their clear regenerative advantages, their clinical success could be compromised by the presence of microbial pathogens. Consequently, there is an urgent clinical need to supplement the inherent bioactivity of collagen with advanced antimicrobial agents. This work introduces a novel class of light‐activatable, NPs‐enriched Coll sheets meant to be used for wound healing applications. By combining the regenerative capabilities of equine‐derived type I Coll with the antibacterial potential of Z‐based NPs, particularly the ZSA type, a multifunctional dressing with the potential to prevent infections and accelerate healing processes was developed. With the objective of taking a step toward the translational validation of Coll/NPs sheets intended for wound care applications, the primary aim of the present study was their development and in vitro proof‐of‐concept validation, with a specific focus on materials engineering, physicochemical characterization, cytocompatibility, and antibacterial performance evaluation under controlled conditions. All data supported that coll sheets structure and bioactivity after NPs integration were preserved, as well as their physico‐chemical mechanical properties, with tuneable degradation kinetics. Antibacterial tests confirmed the effectiveness of Coll/NPs sheets against *E. coli* and *S. aureus* and their enhanced antibacterial behavior under laboratory ambient‐light exposure compared with dark conditions. Results reported support the feasibility of developing light‐modulated antibacterial collagen‐based composites for wound care‐oriented applications. These findings provide the necessary functional framework required before progression to in vitro and in vivo wound healing models. Future investigations will focus on expanding the concentration range and performing detailed dose–response analyses to identify the optimal window for synergistic antimicrobial and regenerative performance. Then, future investigations will prioritize translational and clinically relevant validation steps, including extended cytocompatibility testing on primary human cells, hemocompatibility, inflammatory response profiling, and in vivo evaluation in acute and chronic wound models. Further investigations may explore the integration of bioactive peptides into the collagen matrix to enhance immunomodulatory, angiogenic, or pro‐migratory responses, in line with emerging peptide‐driven strategies for skin regeneration [60, 61, 62, 63, 64]. Particular attention will be devoted to performance assessment in clinically representative conditions, such as diabetic or infected wound models, to verify long‐term antimicrobial persistence and tissue integration [60, 61, 62, 63, 64]. From a regulatory perspective, further studies will address compliance with medical device standards for advanced wound care products.

## Author Contributions

L.S., and S.B. led the Conceptualization. Data curation was performed by N.G., A.N., G.I., F.R., and A.P. Formal analysis was carried out by N.G., F.R., G.I., A.P., and G.E.D. Funding was acquired by A.Q., L.C., and L.S. The Investigation was conducted by N.G., A.N., G.I., S.B., F.R., and A.P. Methodology was developed by N.G., L.C., and G.E.D. Project administration was handled by L.S. and A.S. Resources were provided by A.Q., L.C., L.S., and A.S. Software was developed by N.G., G.I., F.R., and A.N. Supervision was provided by S.B., L.C., L.V., L.S., and A.S. Validation was performed by N.G., L.C., G.E.D. Visualization was carried out by N.G. and G. I. The original draft was written by N.G., G.I., A.N., and the manuscript was reviewed and edited by S.B., A.Q., L.C., L.V., and A.S.

## Funding

This work received no external funding.

## Conflicts of Interest

The authors declare no conflicts of interest.

## Supporting information




**Supporting File**: mabi70188‐sup‐0001‐SuppMat.docx.

## Data Availability

The experimental data used to support the findings of this study are available from the corresponding author upon request.
