# Supplementary material for: Development and In Vitro Characterization of Light Responsive Zinc‐Based Nanoparticles Embedded in Collagen Sheets Intended for Wound Care Oriented Applications
Source: Macromol Biosci. 2026 Apr 27;26:e70188. doi: 10.1002/mabi.70188 (PMC13113222; doi:10.1002/mabi.70188)
Supplement: Supplementary file 1 — Supporting File: mabi70188‐sup‐0001‐SuppMat.docx. [file MABI-26-e70188-s001.docx]

**Supplementary Material**


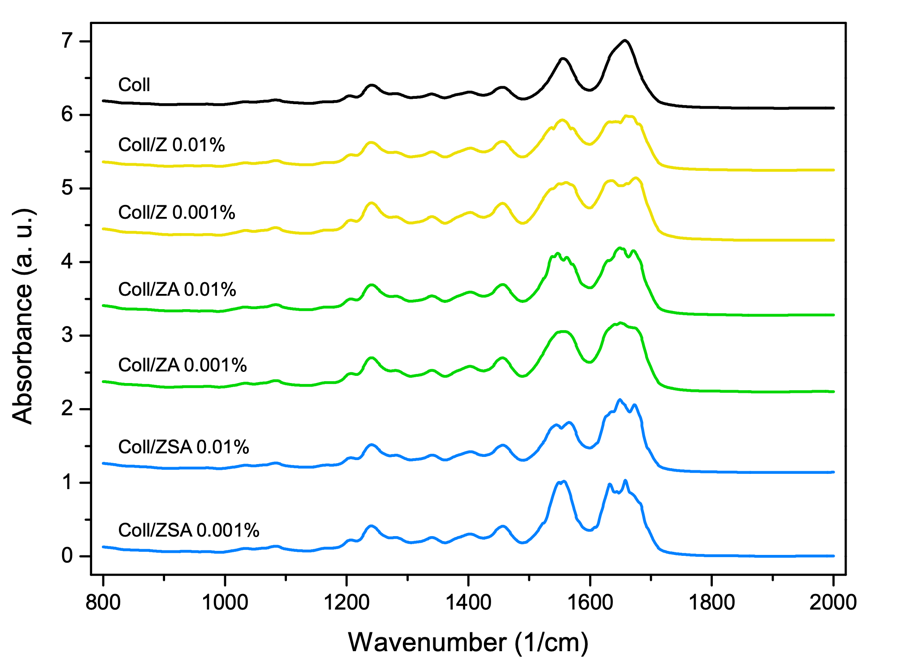


**
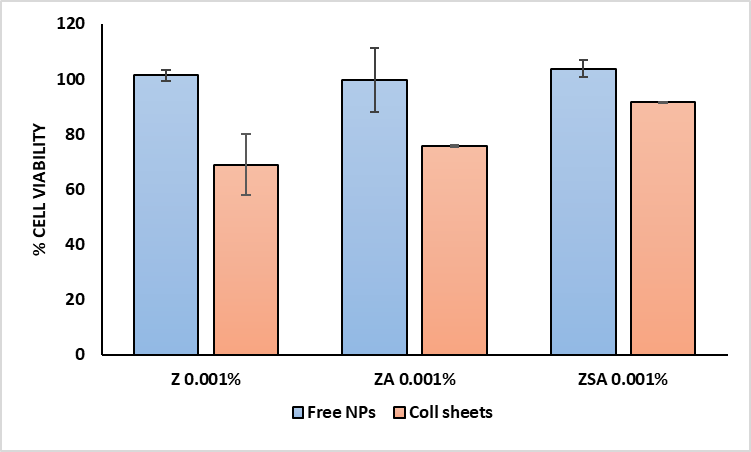
Figure S1.** FT-IR spectra of Coll/NPs sheets in comparison with Coll sheet.

**Figure S2.** Cell viability, determined by MTT assay, of the fibroblasts incubated with either bare nanoparticles or Coll/NPs sheets.

**
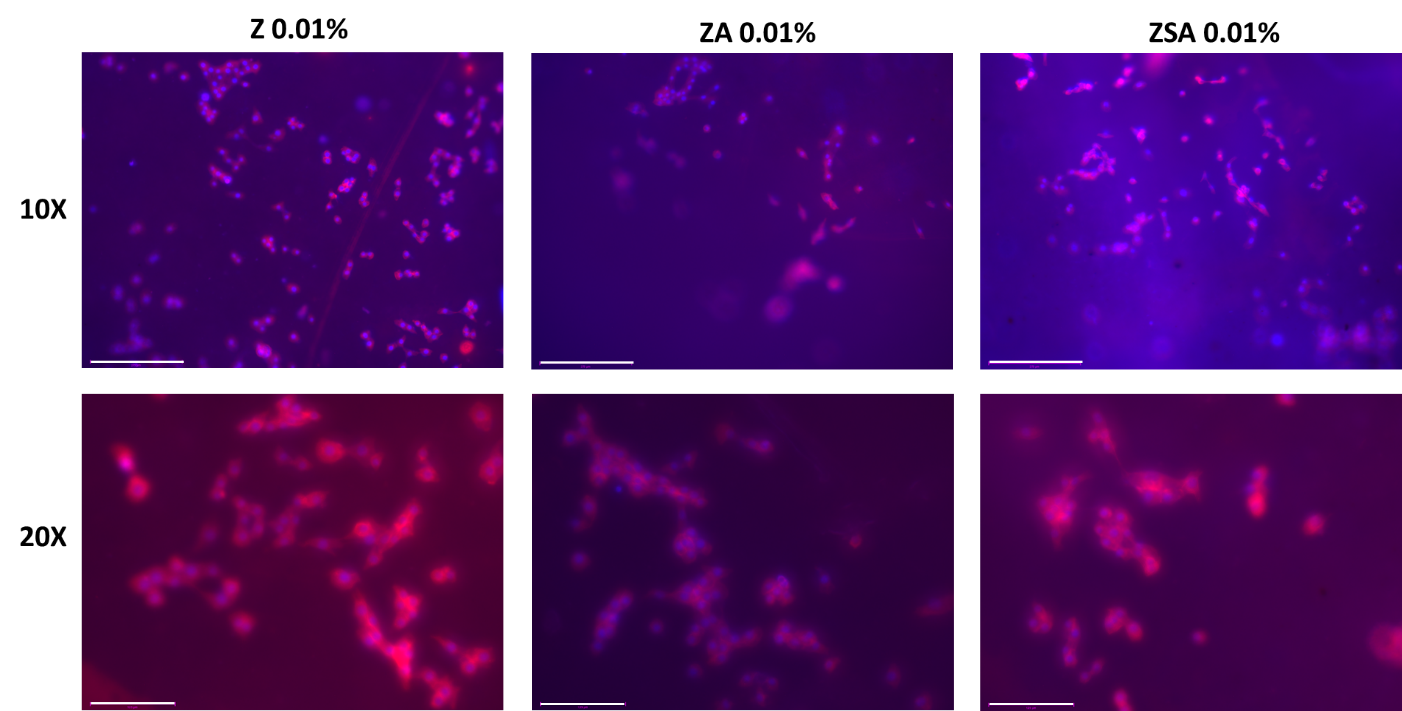
**

**Figure S3**. Fluorescent images of NIH/3T3 grown on Coll/NPs 0.01% sheets for 96h. Scale bar is 275 µm (upper panels) and 125 µm (lower panels).


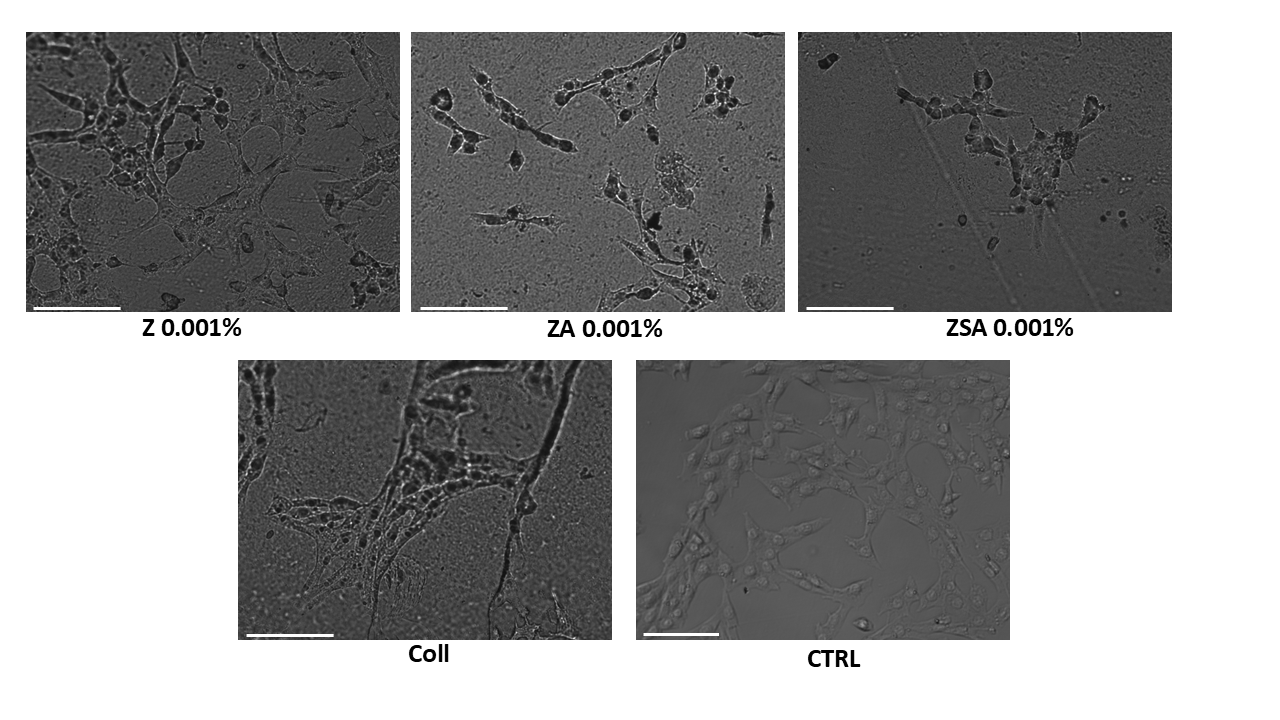


**Figure S4**. Optical images of NIH/3T3 grown on Coll and Coll/NPs 0.001% sheets for 96 h. Scale bar is 100 µm.
